# Ectopic Expression of *Perilla frutescens WRI1* Enhanced Storage Oil Accumulation in *Nicotiana benthamiana* Leaves

**DOI:** 10.3390/plants12051081

**Published:** 2023-02-28

**Authors:** Semi Kim, Kyeong-Ryeol Lee, Mi Chung Suh

**Affiliations:** 1 Department of Life Science, Sogang University, Seoul 04107, Republic of Korea; 2 Department of Agricultural Biotechnology, National Institute of Agricultural Sciences, Jeonju 54875, Republic of Korea

**Keywords:** *Perilla frutescens*, WRINKLED1, transcription factor, triacylglycerol, seed oil

## Abstract

Vegetable oils are indispensable in human and animal diets and have been widely used for the production of detergents, lubricants, cosmetics, and biofuels. The seeds of an allotetraploid *Perilla frutescens* contain approximately 35 to 40% oils with high levels of polyunsaturated fatty acids (PUFAs). WRINKELD1 (WRI1) encoding an AP2/ERF-type transcription factor is known to upregulate the expression of genes involved in glycolysis and fatty acid biosynthesis and TAG assembly. In this study, two *WRI1* isoforms, *PfWRI1A,* and *PfWRI1B* were isolated from Perilla and predominantly expressed in developing Perilla seeds. The fluorescent signals from *PfWRI1A:eYFP* and *PfWRI1B:eYFP* driven by the CaMV 35S promoter were detected in the nucleus of the *Nicotiana benthamiana* leaf epidermis. Ectopic expression of each of *PfWRI1A* and *PfWRI1B* increased the levels of TAG by approximately 2.9- and 2.7-fold in *N. benthamiana* leaves and particularly, the enhanced levels (mol%) of C18:2, and C18:3 in the TAGs were prominent with the concomitant reduction in the amounts of saturated fatty acids. The expression levels of *NbPl-PKβ1*, *NbKAS1*, and *NbFATA*, which were known to be target genes of *WRI1,* significantly increased in tobacco leaves overexpressing *PfWRI1A* or *PfWRI1B*. Therefore, newly characterized *PfWRI1A* and *PfWRI1B* can be potentially useful for the enhanced accumulation of storage oils with increased PUFAs in oilseed crops.

## 1. Introduction

Plants accumulate triacylglycerols (TAGs), carbon, and energy storage components during seed or fruit development [1]. Storage oils produced from oil palm trees (*Elaeis guineesis*), soybean (*Glycine max*), corn (*Zea mays*), and rapeseed (*Brassica napus*) have been used for the production of edible oils and as industrial raw materials for manufacturing paints, detergents, lubricants, and cosmetics [2,3]. Recently, the usage of sustainable vegetable oils has been expanded tremendously to dissolve global warming caused by rapid human population growth and the increased ratio of carbon dioxide emitted from the consumption of large-scale fossil fuels [4,5]. To meet the increased demand for vegetable oils, oilseed crops with enhanced storage oil contents have been developed using genetic engineering technologies [6,7,8,9,10]. Therefore, it is a critical step to isolate novel genetic resources to contribute to the enhancement of TAG levels in oilseed crops.

Fatty acid biosynthesis exclusively occurs in the plastids [11,12]. When pyruvate is produced from glucose through glycolysis, the pyruvate is converted to acetyl-CoA by pyruvate dehydrogenase complex (PDHC) by a process called pyruvate decarboxylation [13]. The PDHC contains pyruvate dehydrogenase α and β subunits (α-PDH and β-PDH), dihydrolipoamide acetyltransferase (DHLAT), and dihydrolipoamide dehydrogenase (DPD). The generated acetyl-CoA is converted to malonyl-CoA by the acetyl-CoA carboxylase (ACCase), which contains carboxyltransferase α and β subunit (α-CT and β-CT), biotin carboxylase (BC), and biotin carboxyl carrier protein (BCCP) [14,15]. Malonyl-CoA is converted to malonyl-acyl-carrier-protein (ACP) by malonyl-CoA: ACP malonyltransferase (MCAMT) [16]. Notably, 3-ketoacyl-ACP is formed through a condensation reaction between malonyl-ACP and acetyl-CoA catalyzed by 3-ketoacyl-ACP synthase III (KAS III) [17]. After that, it undergoes a series of two-carbon elongation reactions by the fatty acid synthase complex to C16 or C18 fatty acyl-ACPs. Fatty acyl-ACP thioesterases (FATA/FATB) hydrolyze the thioester bond to release the fatty acyl group from ACP [18]. The generated C16 and C18 fatty acids are exported to the cytosol in a form of fatty acyl-CoAs by long-chain acyl-CoA synthetases (LACS) [19].

For TAG biosynthesis in the endoplasmic reticulum (ER), the C16 and C18 fatty acyl-CoAs are esterified to the glycerol backbone through the Kennedy or glycerol-3-phosphate pathway [16]. Glycerol-3-phosphate acyltransferase (GPAT) causes the first acylation at *sn*-1 of G3P to produce lysophosphatidic acid (LPA), and then the second acylation of an acyl-CoA is processed by 2-lysophosphatidic acid acyltransferase (LPAAT) to *sn*-2 of LPA to form phosphatidic acid (PA). Next, diacylglycerol (DAG) is produced by the dephosphorylation of PA via phosphatidate phosphatase (PAP). Finally, DAG acyltransferase (DGAT) generates TAG by acylation of *sn*-3 of DAG using a fatty acyl-CoA. DAG is also acylated by phospholipid: diacylglycerol acyltransferase (PDAT) using phosphatidyl choline (PC) as an acyl donor [20,21,22]. In addition, TAG is also synthesized by a cytosolic DGAT-dependent pathway, which is sequentially catalyzed by soluble GPAT, LPA phosphatase, monoacylglycerol acyltransferase (MGAT), and DGAT3 [23,24,25,26,27]. 

It was first reported that the Arabidopsis *wrinkled1* (*wri1*) mutant impaired starch accumulation, seed germination, seedling development, and storage oil deposition [28]. Later, Arabidopsis WRI1, which belongs to the plant-specific APETALA2/ethylene-responsive element binding factor (AP2/ERF) family was identified to upregulate the expression of genes involved in glycolysis and fatty acid biosynthesis by directly binding to the promoters of pyruvate kinase β subunit 1 (*PI-PKβ1*), pyruvate dehydrogenase (*PDHE1α*), biotin carboxyl carrier protein 2 (*BCCP2*), and *KAS I* [29,30,31,32,33,34,35,36]. The reduced oil content in Arabidopsis *wri1* mutants was rescued to the wild-type levels by the expression of several *WRI1* orthologs isolated from *Zea mays*, camelina (*Camelina sativa*), jatropha (*Jatropha curcas*), *Elaeis guineensis*, and *Glycine max* [34,35,36,37,38,39]. In Arabidopsis, camelina, and rapeseed (*Brassica napus*) seeds overexpressing *AtWRI1 or BnWRI1*, the oil content increased by approximately 7 to 20% compared with the control [37,38,40,41,42,43,44]. When *Arabidopsis*, potato (*Solanum tuberosum*), oat (*Avena sativa*), nutsedge (*Cyperus esculentus*), or poplar (*Populus trichocarpa*) *WRI1* was transiently expressed in *Nicotiana benthamiana* leaves, the levels of TAGs was also elevated [37,42].

Annual herbaceous seed oil crop, *Perilla frutescens* belongs to the *Lamiaceae* family and contains 35–45% seed storage oils harboring approximately 70 to 80% ω-3 fatty acids (α-linolenic acid, C18:3) and ω-6 fatty acids (linoleic acid, C18:2) [43]. Perilla is an allotetraploid plant (2n = 40), which is a hybrid of *P. citriodora* (2n = 20) and another diploid species (2n = 20) [44,45]. Transcriptome analysis from developing Perilla seeds provided genetic resources involved in storage oil biosynthesis and enabled the functional characterization of oleate desaturase (fatty acid desaturase 2; FAD2) and linoleate desaturase (FAD3), which are involved in the biosynthesis of linoleic and linolenic acids, respectively, and DGAT1 required for TAG biosynthesis [8,46]. Recently, Perilla genome sequencing revealed that the acyl-CoA: lysophosphatidylcholine acyltransferase (*LPCAT*) gene contributes to the high α-linolenic acid content in seed storage oils [47]. 

In this study, we isolated *WRI1A* and *WRI1B* isoforms, which were predominantly expressed in developing Perilla seeds. The fluorescent signals from the *PfWRI1*: *enhanced yellow fluorescence protein* (*eYFP*) construct were localized to the nucleus in the *N. benthamiana* leaf epidermal cells. When the *PfWRI1*:*eYFP* driven by the cauliflower mosaic virus (CaMV) 35S promoter was expressed in *N. benthamiana* leaves, oil body formation was detected by Nile red staining, the levels of TAGs were examined by thin-layer chromatography (TLC) and gas chromatography (GC) and the transcript levels of *NbPl-PKβ1*, *NbKASI*, and *NbFATA* were investigated. This study revealed that *PfWRI1s* harbor the potential for the enhancement of storage oil biosynthesis and can be useful for the development of oilseed crops with increased seed oils containing high levels of linoleic and α-linolenic acids.

## 2. Materials and Methods

### 2.1. Plant Materials and Growth Conditions

*N. benthamiana* (Nb) and *P. frutescens* var. *frutesces* (*Pf*) were used in this study. Seeds were sown in the soil (soil: perlite = 3:1) and then plants were grown in the growth condition, which was maintained at 23 ± 2 °C, 50–60% humidity, and light and dark cycles (16 h/8 h, L/D). 

### 2.2. Isolation and Subcellular Localization of PfWRI1A and PfWRI1B

Total RNA was isolated from developing Perilla seeds 2 to 3 weeks after flowering by modifying the method of [48]. Total RNA was extracted from the finely ground developing seeds under liquid nitrogen in extraction buffer (8 M LiCl, 1 M Tris-HCl, pH 8.0, 0.5 M EDTA, and 10% sodium dodecyl sulfate) and chloroform, cleaned up by phenol:chloroform: isoamyl alcohol (25:24:1 = *v/v/v*) extraction, and precipitated in 8 M LiCl at 4 °C. DNase I (Qiagen, Hilden, Germany)-treated RNAs were converted to cDNAs in GoScript^TM^ 5X reaction buffer, 2.5 mM MgCl_2_, 0.5 mM dNTP, and GoScript^TM^ reverse transcriptase according to the manufacturer protocol (Promega, Madison, WI, USA). The generated cDNAs were used for *PfWRI1* cDNA isolation, reverse transcription-polymerase chain reaction (RT-PCR), and RT-quantitative PCR analyses.

To isolate *PfWRI1A* and *PfWRI1B* cDNAs and further examine their subcellular localization, the generated cDNAs were amplified by PCR using SacI-PfWRI1_F1 and XmaI-PfWRI1_R1 primers (Supplementary Table S1). The amplified *PfWRI1s* PCR products were digested with *Sac*I (5′ terminus) and *Xma*I (3′ terminus) and then cloned into the binary vector pPZP212. Two WRI1 isoforms, *PfWRI1A,* and *PfWRI1B* were identified after sequencing of the clones. *PfWRI1s* were translationally fused with eYFP in the pPZP212 vector and expressed under the control of the CaMV promoter. Subsequently, the binary constructs were transformed into *Agrobacterium tumefaciens* GV3101 using the freeze-thaw method [49]. The transformed Agrobacteria suspended in infiltration media (10 mM MES, pH5.7, 10 mM MgCl_2_, 200 µM acetosyringone) were infiltrated into *N. benthamiana* leaves. The infiltrated leaves were observed using a confocal laser scanning microscope (Leica TCS SPE, Weitzlar, Germany) 48 h after infiltration. The YFP fluorescence signal was observed at 488 nm excitation and 532 nm emission wavelengths. To visualize the nucleus in the infiltrated leaves, the leaves were stained with DAPI solution (5 µg/mL in PBS) for 5 min, washed 1–2 times with DW, and then the fluorescent signals were obtained at 359 nm excitation and 461 nm emission. 

### 2.3. Protein Sequence Alignment and Phylogenetic Tree Analysis of WRI1 Orthologs from Various Plant Species

The deduced amino acid sequence similarity of PfWRI1A, PfWRI1B, and AtWRI1 was analyzed using Multiple Sequence Alignment by CLUSTALW (http://www.genome.jp/tools/clustalw/ (accessed on 25 May 2018) and GeneDoc (https://genedoc.software.informer.com/) programs. Phylogenetic tree analysis of interspecies obtained through Basic Local Alignment Search Tool (http://blast.ncbi.nlm.nih.gov/Blast.cgi (accessed on 17 March 2022) and Phytozome (https://phytozome-next.jgi.doe.gov/) was performed. Dendrograms were constructed using MEGA X software to prepare a phylogenetic tree [50]. The Bootstrap method was set to 1000 replicas.

### 2.4. RT-PCR and RT-qPCR Analyses

To investigate the expression of *PfWRI1s* in various Perilla organs, total RNA was extracted from leaves, stems, roots, and open flowers of approximately 3-month-old Perilla plants using a total RNA isolation kit (Qiagen). RNA was reverse transcribed into cDNA in the same manner as described above, and then the generated cDNA was subjected to the RT-PCR and RT-qPCR analyses using gene-specific primers (Table S1). RT-PCR was performed in a volume of 20 μL with Prime Taq Premix (GENET BIO, Daejeon, Republic of Korea); 1 cycle at 94 °C for 5 min, 28 cycles at 94 °C for 30 s, 61 °C for 30 s, and 72 °C for 30 s, and 1 cycle at 72 °C for 7 min. RT-qPCR was performed in a volume of 20 μL with TOPreal^TM^ SYBR Green qPCR PreMix (Enzynomics, Daejeon, Republic of Korea); 1 cycle at 95 °C for 12 min, 30 cycles at 95 °C for 20 s, 60 °C for 20 s, 72 °C for 20 s, and 1 cycle at 95 °C for 10 s, 65 °C for 60 s, 97 °C for 1 s (CFX96 real-time PCR system, Bio-Rad, Heracles, CA, USA).

To examine the expression of *PfWRI1s* and their target genes in *N. benthamiana* leaves expressing *PfWRI1A* or *PfWRI1B*, total RNA was isolated from the transformed leaves using a total RNA isolation kit (Qiagen) and gene-specific primers of *NbACT*, *NbBCCP*, *NbKAS1*, *NbENR1*, *NbFATA*, *NbPI-PKβ1*, and *NbPDH-E1α* genes were designed by BLAST searches of the *N. benthamiana* genome sequence database (Sol Genomics Network, https://solgenomics.net/organism/Nicotiana_benthamiana/genome (accessed on 26 November 2022) [51]) using their Arabidopsis orthologs as queries and based on the previous report (Supplementary Table S1) [52]. RT-PCR and RT-qPCR were performed as described above with some modifications. *N. benthamiana* Actin 2 gene (Niben101Scf06087g02002) was used to determine quantity and quality of cDNAs.

### 2.5. Transient Expression of PfWRI1A and PfWRI1B in N. benthamiana Leaves and Nile Red Staining

*Agrobacterium* containing *PfWRI1A*:eYFP, *PfWRI1B*:eYFP, or empty vector (pPZP212) were cultured to OD_600_ = 0.8. Each *Agrobacterium* was mixed with *Agrobacterium* cell containing p19 in the infiltration solution, and then infiltrated into 4–5 week-old *N. benthamiana* leaves. The transformed leaves were stained with Nile red solution (10 µg/mL in 0.1 M Tris-HCl, pH 8.0) at room temperature for 30 min in dark conditions. It was washed twice for 5 min with Tris-HCl buffer (pH 8.0), and oil body formation was observed at 560 nm excitation and 615 nm emission wavelength using a confocal laser scanning microscope (TCS SPE, Leica Microsystems, Weitzlar, Germany).

### 2.6. TLC and GC Analyses

Six days after *Agrobacterium* infiltration, *N. benthamiana* leaves were finely ground under liquid nitrogen and freeze-dried and then used for TLC analysis. The dry residues (~5 mg) were mixed with glyceryl triheptadecanoate (C17:0) internal standard (15 μg/mL) in 1.5 mL of chloroform: methanol (2:1 = *v/v*) by vigorous vortexing and then mixed with 500 μL of 0.1 M KCl. After centrifugation, the lower lipid phase was concentrated under nitrogen gas and dissolved in 100 μL of chloroform. The extracted lipids were loaded on a TLC plate (Kieselgel 60, MERC) and separated in hexane: diethyl ether: acetic acid (70:30:1, *v/v/v*). After the TLC plate was sprayed with 80% acetone containing 0.01% primuline, the bands corresponding to TAGs were visualized under UV light. The marked TAG bands were scraped off, mixed with toluene and 1 mL of 5% H_2_SO_4_ in methanol, and then methyl-esterified at 90 °C for 90 min. After 1.5 mL of aqueous 0.9% NaCl (*w/v*) was added, fatty acid methyl esters (FAMEs) were extracted with 2 mL of hexane three times. The concentrated FAMEs were analyzed on a GC-2010 (Shimadzu, Kyoto, Japan) equipped with flame ionization detector (FID). DB-23 column (30 mm × 0.25 mm, 0.25 μm film thickness; J&W Scientific, Folsom. CA, USA) was used and GC conditions were set to increase at a rate of 2.5 °C per minute from 160 °C to 220 °C. For each FAMEs, retention time and peak areas of internal standards were compared and analyzed. The composition and amount of FAMEs were analyzed by comparison of their retention times and peak areas with those of internal and individual standards.

## 3. Results

### 3.1. Nucleotide and Amino Acid Sequences of P. frutescens WRI1 Isoforms and Phylogenetic Tree in WRI1 Orthologs from Various Plant Resources

**Figure 1:**
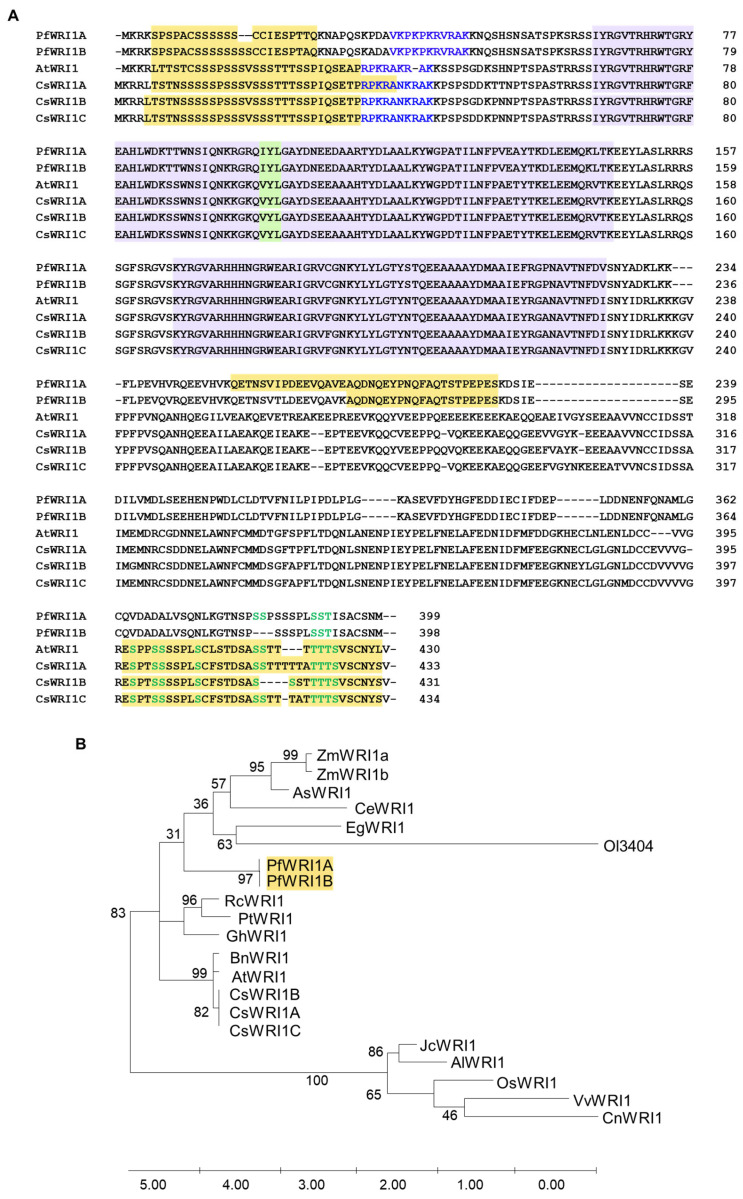
Alignment of the deduced amino acid sequences of *WRI1* isoforms from *P. frutescens*, *A. thaliana*, and *C. sativa* and phylogenetic tree of *PfWRI1* homologs from diverse plant species. (**A**) Whitish purple and green backgrounds indicate the conserved AP2/ERF DNA binding motifs and the transcriptional activation motif in WRI1, respectively. The predicted nuclear localization signals (NLS) sequences of AtWRI1A (PRPKRAKRAK, 334−42 aa), PfWRI1A (VKPKPKRVRAK, 36−46 aa), PfWRI1B (VKPKPKRVRAK, 38−48 aa), and CsWRI1A to C (RPKRANKRAK, 35−43 aa) are shown in blue letters. PEST motifs were analyzed by ePESTfind (https://emboss.bioinformatics.nl/cgi-bin/emboss/epestfind (accessed on 1 June 2000) and are shown in yellow backgrounds. Ten phosphorylation sites of Arabidopsis WRI1 are involved in its regulatory function [53], and their putative corresponding sites in PfWRI1s and CsWRI1s are shown in green letters. (**B**) Phylogenetic tree of *PfWRI1* homologs from diverse plant species. The phylogenetic tree was generated using MEGA X by the maximum likelihood method. Bootstrap value percentages of 1000 replicates are shown at the branching points. *AtWRI1*, *Arabidopsis thaliana* (824599); *PfWRI1s*, *Perilla frutescens* var. *frutescens in this study*; *AsWRI1*, *Avena sativa* (SRX1079426); *BnWRI1*, *Brassica napus* (ABD16282.1); *CsWRI1A*, *CsWRI1B* and *CsWRI1C*, *Camelina sativa*; *EgWRI1*, *Elaeis guineensis*; *GhWRI1*, *Gossypium hirsutum* (TC200263); *OsWRI1*, *Oryza sativa* (CAE00853.1); *PtWRI1*, *Populus trichocarpa* (SRX1079428); *Ol34044*, *Ostreococcus lucimarinus* (34044); *RcWRI1*, *Ricinus communis* (AB774159.1, AB774160.1); *ZmWRI1*, *Zea mays* (ACF83189.1, ACF80269.1); *JcWRI1*, *Jatropha curcas* (AIA57945.1); *AlWRI1*, *Arabidopsis lyrata* (EFH52510.1); *VvWRI1*, *Vitis vinifera* (CBI32013.3); *CnWRI1*, *Cocos nucifera* (JQ040545).

To isolate *WRI1* orthologs in *P. frutescens*, total RNAs were isolated from developing Perilla seeds and then the converted cDNAs were subjected to the RT-PCR using *PfWRI1* gene-specific primers (Supplementary Table S1). When the PCR products were sequenced, two 1200-nucleotide-long and 1197-nucleotide-long *WR1* isoforms were isolated and named *PfWRI1A* and *PfWRI1B*, respectively (Figure S1). The nucleotide sequence similarity between *PfWRI1A* and *PFWRI1B* was very similar (about 97%), suggesting that the two isoforms are very well conserved (Figure S2). Next, the deduced amino acid sequences of *PfWRI1A* and *PfWRI1B* were compared with those of *WRI1* orthologs from various plant resources including an Arabidopsis *WRI1,* and their phylogenetic tree was constructed. As shown in an Arabidopsis *WRI1*, both PfWRI1A and PfWRI1B harbor two AP2/EREBP DNA binding motifs and an ‘IYL’ motif instead of ‘VYL’, which is known to be a transcriptional activation motif in AtWRI1 (Figure 1A and Figure S2) [32]. The 17th and 18th serine residues in PfWRI1B were deleted in PfWRI1A, whereas thee amino acid residues corresponding to proline, serine, and serine at 384th to 386th in PfWRI1A were not present in PfWRI1B (Figure 1A). The proline and serine motifs (or PEST motifs), which were reported to be involved in the regulation of WRI1 [53] were searched by ePESTfind (https://emboss.bioinformatics.nl/cgi-bin/emboss/epestfind (accessed on 1 June 2000) and then the PEST motifs were detected in the N- and C-terminal regions of Arabidopsis and Camelina WRI1s, but in the middle region of Perilla WRI1A and WRI1B (Figure 1A). The putative amino acid residues, which correspond to the phosphorylation sites in the PEST motifs of AtWRI1 [53] were also shown in PfWRIs and CsWRI1s (Figure 1A), but their regulatory roles in PfWRIs and CsWRI1s remain to be further investigated. In the search of plant WRI1s, we found that one *WRI1* isoform was detected from various crops examined, except *Zea may*s (*ZmWRI1a* and *ZmWRI1b*) and *Camelina sativa* (*CsWRI1 A*, *B,* and *C*). In the phylogenetic tree of plants, *PfWRI1s* and the clade including ZmWRI1, *Avena sativa* AsWRI1, *Cyperus esculentus* CeWRI1, EgWRI1, and *Ostreococcus lucimarinus* Ol3404 were divided after the clade containing *Brassica WRI1s* were divided from the ancestor (Figure 1B). The deduced amino acid sequence similarity of WRIs orthologs from various plant species are shown in Supplementary Table S2.

### 3.2. Expression of PfWRI1A and PfWRI1B in Various Perilla Organs 

**Figure 2:**
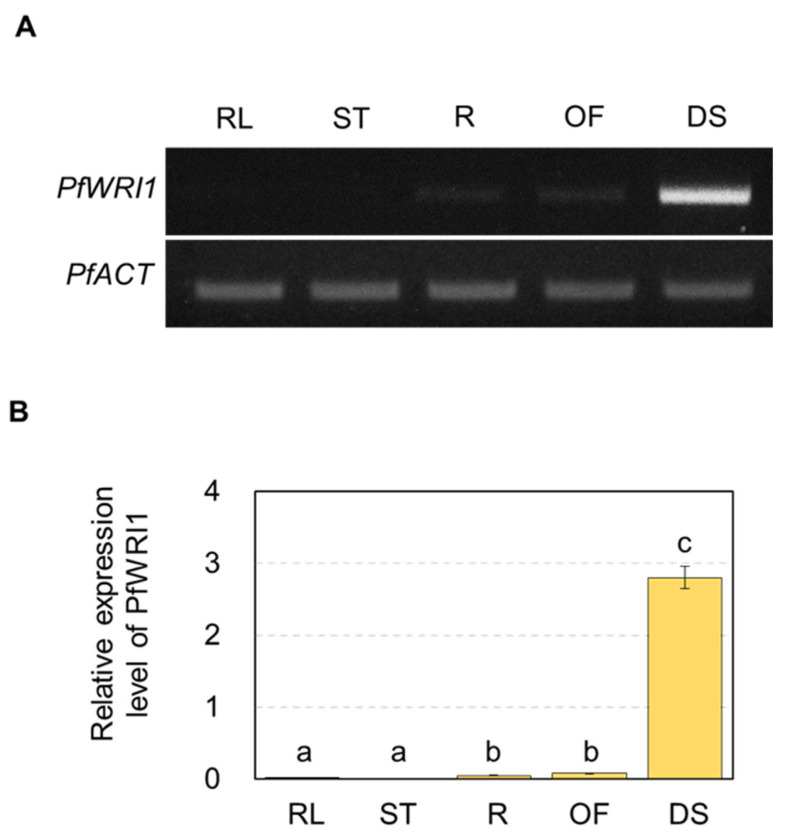
Expression of Perilla *WRI1s* in various *P. frutescens* organs. (**A**) RT-PCR analysis of *PfWRI1s* in various *P. frutescens* organs. Total RNAs were isolated from rosette leaves (RL), stems (ST), roots (R), and open flowers (OF) of 3-month-old Perilla plants and developing seeds (DS) for 2 to 3 weeks after flowering. *P. frutescens ACTIN* (*PfACT*) was used to determine cDNA quantity and quality. (**B**) RT-qPCR analysis of *PfWRIs1* in various *P. frutescens* organs. The *PfACT* was used to normalize the levels of *PfWRI1* transcripts. Values are the means of the ± SE of three replicates. Data were statistically analyzed using ANOVA test (*p* < 0.05).

To examine the transcript levels of *PfWRI1A* and *PfWRI1B* in various Perilla organs, total RNAs were isolated from rosette leaves, stems, roots, open flowers, and developing seeds of *P. frutescens* plants, and subjected to the RT-PCR and RT-quantitative PCR analyses. Both RT-PCR and RT-qPCR analyses revealed that *PfWRI1s* were predominantly ex-pressed in developing Perilla seeds (Figure 2A,B). Initially, we tried to design *PfWRI1A-* or *PfWRI1B*-specific primers. The design of *PfWRI1B-*specific primers was successful, but it failed to design *PfWRI1A-*specific primers due to a high sequence homology between *PfWRI1A* and *PfWRI1B* (Figure S2). Thus, the total transcript levels of *PfWRI1A* and *PfWRI1B* were shown in Figure 2B. 

### 3.3. Subcellular Localization of PfWRI1A:eYFP and PfWRI1B:eYFP in N. benthamiana Leaves

**Figure 3:**
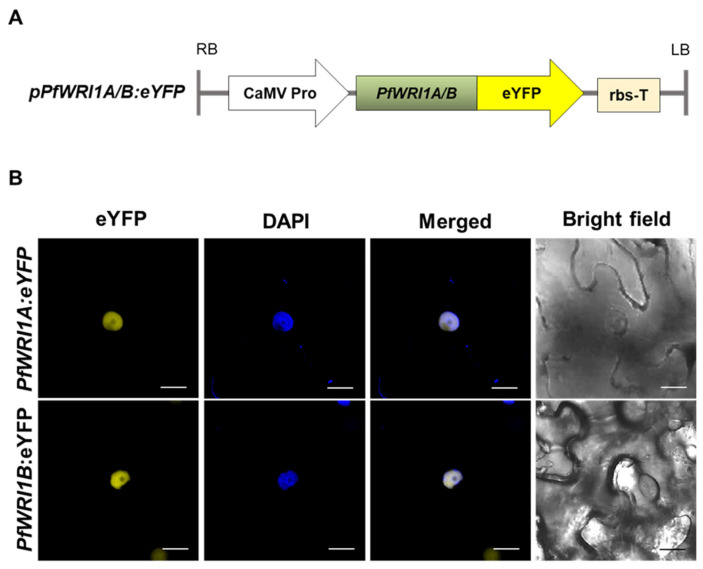
Subcellular localization of *PfWRI1s:eYFP* in *N. benthamiana* epidermis. (**A**) Schematic diagram of *PfWRI1A*:*eYFP* and *PfWRI1B*:*eYFP* constructs. CaMV Pro, cauliflower mosaic virus 35S promoter; LB, left border; RB, right border; rbs-T, the terminator of ribulose-1,5-bisphosphate carboxylase and oxygenase small subunit from pea (*Pisum sativum*). (**B**) Agrobacterium harboring the *PfWRI1A*:*eYFP* (upper) or *PfWRI1B*:*eYFP* (bottom) construct was infiltrated into *N. benthamiana* leaves and then the fluorescent signals were visualized under laser confocal scanning microscopy. The nucleus was visualized by staining with DAPI under the UV filter. The fluorescent signals from eYFP and DAPI were merged. Bright-field images visualized epidermal cells. Bars = 20 µm.

To obtain the binary vector constructs containing *PfWRI1A:eYFP* or *PfWRI1B:eYFP*, the coding regions of the isolated *PfWRI1A* and *PfWRI1B* were fused in-frame eYFP under the control of CaMV 35S promoter in the pPZP212 binary vector (Figure 3A). The *PfWRI1A:eYFP* and *PfWRI1B:eYFP* constructs were transformed into *Agrobacterium* (GV3101 strain), and the transformed Agrobacteria were infiltrated into the *N. benthamiana* leaves. The leaves containing the *Agrobacterium*-infiltrated sites were stained with a DAPI solution 48 h after infiltration and then observed using a confocal laser microscope. The fluorescent signals from both *PfWRI1A:eYFP* or *PfWRI1B:eYFP* constructs were merged with the DAPI signals, indicating that PfWRI1A and PfWRI1B are localized in the nucleus in *N. benthamiana* leaf epidermal cells (Figure 3B) and might play a role as a transcription factor.

### 3.4. Oil Body Formation and TAG Measurement in N. benthamiana Leaves Expressing PfWRI1A or PfWRI1B

**Figure 4:**
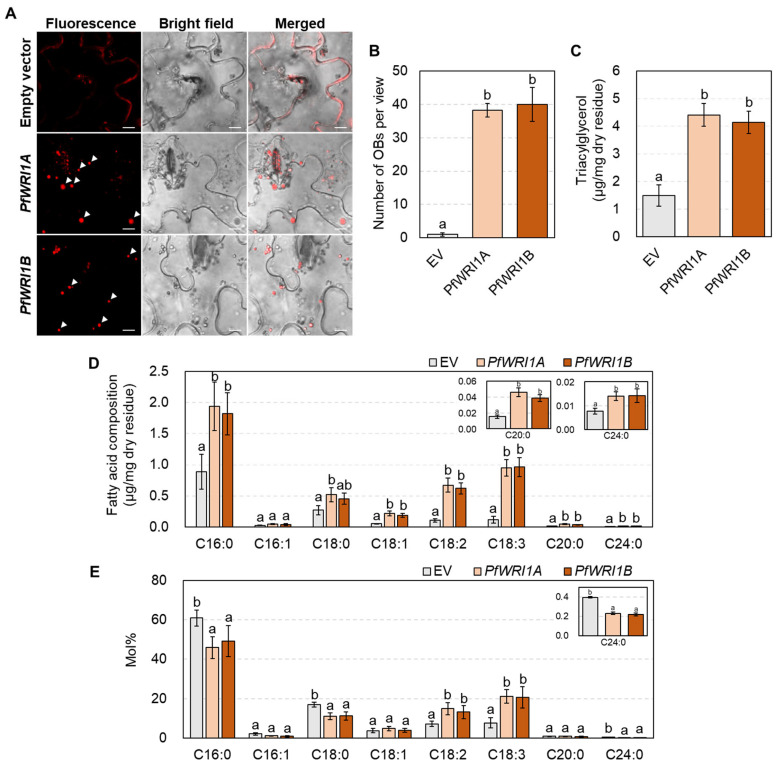
Oil body formation (**A**), oil body counts (**B**) and TAG accumulation (**C**–**E**) in *N. benthamiana* leaves expressing *PfWRI1A:eYFP* or *PfWRI1B:eYFP*. (**A**) Oil body formation in *N. benthamiana* leaves expressing *PfWRI1A*:*eYFP* or *PfWRI1B*:*eYFP.* Agrobacterium harboring the *PfWRI1A:eYFP*, *PfWRI1B:eYFP*, or empty vector without *PfWRI1* construct was infiltrated into *N. benthamiana* leaves. The transformed *N. benthamiana* leaf disks were stained with Nile red solution and then the fluorescent signals were visualized under laser confocal scanning microscopy. The white arrows indicate oil bodies (OBs). Bars = 10 μm. (**B**) Oil body (OB) counts in *N. benthaminana* leaves expressing empty vector (*pPZP212*), *PfWRI1A*, or *PfWIR1B*. Values are averages and SD of four individual images. Data were statistically analyzed using ANOVA test (*p* < 0.05). (**C**–**E**) Total TAG levels (**C**) and fatty acid levels (**D**) and composition (**E**) in the TAG fractions in *N. benthamiana* leaves expressing *PfWRI1A:eYFP*, *PfWRI1B:eYFP*, or empty vector. Total lipids were extracted from the transformed *N. benthamiana* leaf disks and fractionated by thin-layer chromatography. The TAG fractions were eluted, transmethylated, and then the fatty acid methyl esters were analyzed by gas chromatography. Values are averages and SD of four individual experiments. Data were statistically analyzed using ANOVA test (*p* < 0.05).

To investigate the functional activities of *PfWIR1A* and *PfWRI1B*, *Agrobacteria* containing *PfWRI1A:eYFP* or *PfWRI1B:eYFP* driven by the CaMV 35S promoter were infiltrated in *N. benthamiana* leaves. The leaves containing the *Agrobacterium*-infiltrated sites were stained with Nile red solution 6 d after infiltration and then oil bodies were observed under confocal microscopy. Ectopic expression of *PfWIR1A* or *PfWRI1B* caused the formation of oil bodies in *N. benthamiana* leaves, but no signals corresponding to oil bodies were observed in *N. benthamiana* leaves infiltrated with the pPZP212 binary vector without *PfWRI1* (Figure 4A). The number of oil bodies significantly increased in the leaves expressing *PfWIR1A* or *PfWRI1B* relative to the control, indicating that the *PfWRI1A* and *PfWRI1B* may contribute to the accumulation of storage oils in *N. benthamiana* leaves (Figure 4B).

Subsequently, the amount of TAGs accumulated in the oil bodies in *N. benthamiana* leaves expressing *PfWRI1A* or *PfWRI1B* was measured by TLC and GC with a flame ionization detector. Briefly, the *N. benthamiana* leaves expressing *PfWRI1A* or *PfWRI1B* were lyophilized and then subjected to the extraction of total lipids. After the chloroform-extracted lipids were separated by TLC and visualized with 0.01% primuline under UV light (Supplementary Figure S3), the bands corresponding to the TAGs were eluted, and then further analyzed by GC. The levels of total FAMEs in *N. benthamiana* leaves expressing *PfWRI1A* and *PfWRI1B* increased approximately 2.9- and 2.7-fold relative to the control transformed with the *pPZP212* binary vector without *PfWRI1*, respectively (Figure 4C). In fatty acid composition analysis, the levels of all fatty acid components except C16:1 significantly increased in the leaves expressing *PfWRI1A:eYFP* or *PfWRI1B:eYFP* compared with the control expressing *eYFP* (Figure 4D). The ratio of polyunsaturated fatty acids, C18:2 and C18:3 were elevated by approximately 2- and 3-fold, but the proportions of saturated fatty acids, C16:0, C18:0, and C24:0 decreased by approximately 22%, 34%, and 50% in *N. benthamiana* leaves expressing *PfWRI1A* and *PfWRI1B* compared with the control, respectively (Figure 4E). Taken together, ectopic expression of each of *PfWRI1A* and *PfWRI1B* enhanced the levels of TAG with unsaturated fatty acids (C18:2, and C18:3) in *N. benthamiana* leaves.

### 3.5. Induced Expzression of WRI1′s Target Genes in N. benthamiana Leaves Expressing PfWRI1A or PfWRI1B

**Figure 5:**
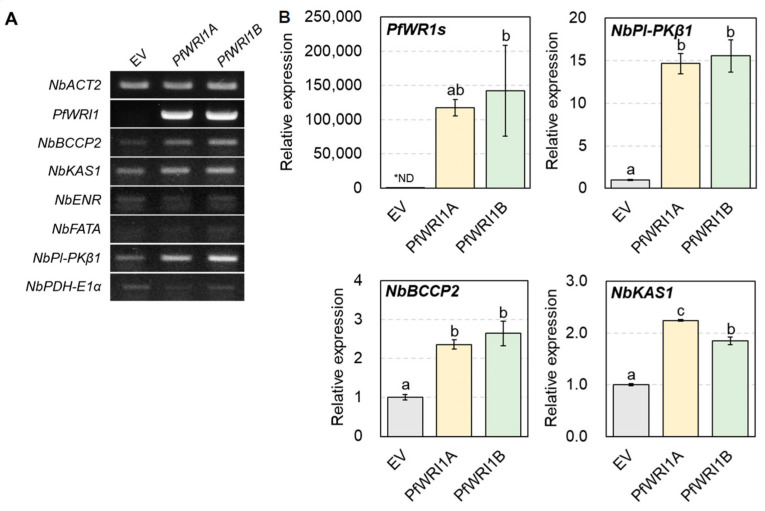
Expression of its target genes in *N. benthamiana* leaves expressing *PfWRI1A* or *PfWRI1B*. (**A**) RT-PCR analysis of its target genes in *N. benthamiana* leaves expressing *PfWRI1A* or *PfWRI1B.* Total RNA was isolated from *N. benthamiana* leaves expressing *PfWRI1A*, *PfWRI1B*, or empty vector control and converted to the cDNA, which was subjected to the RT-PCR analysis. *N. benthamiana Actin2* was used to determine cDNA quantity and quality. (**B**) RT-qPCR analysis of its target genes in *N. benthamiana* leaves expressing *PfWRI1A* or *PfWRI1B. NbActin2* was used to normalize the levels of *PfWRI1s* and their target genes’ transcripts. Each value is the mean of three independent measurements ± standard error. Data were statistically analyzed using ANOVA test (*p* < 0.01). * ND indicates non-detected.

To investigate whether or not the ectopic expression of *PfWRI1A* and *PfWRI1B* upregulates the expression of WRI1’s target genes, total RNAs were extracted from *N. benthamiana* leaves expressing *PfWRI1A* or *PfWRI1B* 2 d after *Agrobacterium* infiltration and converted to the cDNAs. The synthesized cDNAs were subjected to RT-PCR and RT-qPCR analyses using the gene-specific primers, which were searched from the *N. benthamiana* transcriptome database (https://solgenomics.net/organism/Nicotiana_benthamiana/genome (accessed on 26 November 2014). *N. benthamiana* leaves transformed with the *pPZP212* binary vector without *PfWRI1* were used as a control. In RT-PCR analysis, we observed the induced expression of *NbBCCP2*, *NbKAS1*, and *NbPl-PKβ1 in N. benthamiana* leaves expressing *PfWRI1A* or *PfWRI1B* relative to the control, but no significant differences in the levels of *NbENR*, *NbFATA*, and *NbPDH-E1α* transcripts were observed between *N. benthamiana* leaves expressing *PfWRI1A* or *PfWRI1B* and the control leaves (Figure 5A). We further measured the levels of *PfWRI1*, *NbBCCP2*, *NbKAS1*, and *NbPl-PKβ1* transcripts by RT-qPCR. The *NbACT* was used for the determination of the quantity and quality of cDNAs. As shown in Figure 5B, the expression of *NbPl-PKβ1* transcripts was upregulated by approximately 15- and 16-fold in *N. benthamiana* leaves expressing *PfWRI1A* and *PfWRI1B* relative to the control, respectively. The expression of *NbBCCP2* and *NbKAS1* was also elevated by approximately two- to three-fold in *N. benthamiana* leaves expressing *PfWRI1A* or *PfWRI1B* compared with the control. The results revealed that *PfWRI1A* and *PfWRI1B* are able to accumulate storage oils in *N. benthamiana* leaves by the upregulation of their target genes such as *NbBCCP2*, *NbKAS1*, and *NbPl-PKβ1.*

## 4. Discussion

Since the usage of vegetable oils has increased tremendously as a sustainable and alternative energy resource, it is known to be the critical step in elevating oil content in oilseed crops [2,3,4]. Vanhercke et al. [54] reported that the “Push-Pull-Protect” module was suggested to increase storage oil deposition in genetically engineered seeds or leaves by an increase in the production of fatty acids in the plastids (Push), an increase in TAG assembly and accumulation (Pull), and a decrease in TAG hydrolysis or catabolism (Protect). WRINKLED1 (WRI1) is well known as a “Push” factor to activate fatty acid biosynthesis by the upregulation of genes involved in glycolysis and fatty acid biosynthesis and thereby enhances TAG accumulation [28,29,30]. In this study, we identified a noble genetic resource, *P. frutescens WRI1* genes, which contribute to the increased accumulation of storage oils in *N. benthamiana* leaves.

Since the *WRI1* was first reported from *Arabidopsis* (Cernac and Benning, 2004), several *WRI1* orthologs have been characterized from various monocot and dicot plants including *Brassica napus* [55], *Camelina sativa* [37], *Zea mays* [35,56], *Jatropha curcas* [38], and *Oryza sativa* [57]. In the previous report [42], the *WRI1* orthologs from Arabidopsis (*AtWRI1*), potato (*StWRI1*), oat (*AsWRI1*), poplar (*PtWRI1*), and nutsedge (*CeWRI1*) were transiently expressed in tobacco leaves and then a significant increase in TAG accumulation was observed in all transformed leaves relative to the control. Ectopic expression of *C. sativa WRI1A*, *B*, or *C* induced the formation of oil bodies in *N. benthamiana* leaves and eventually increased TAG levels by approximately 2.5- to 4.0-fold in the leaves compared to the control [37]. The formation of oil bodies was also observed in *N. benthamiana* leaves expressing *PfWRI1A* or *PfWRI1B* by the Nile red staining (Figure 4A). In the tobacco leaves expressing *PfWRI1A* and *PfWRI1B*, the levels of TAGs were significantly elevated relative to the control (Figure 4B), indicating that the *PfWRI1A* and *PfWRI1B* enhance storage oil deposition in leaves, which have been used for the production of vegetable oils as an alternative non-seed organ [48]. 

In the fatty acid composition in the TAG fractions accumulated in *N. benthamiana* leaves expressing *AtWRI1*, *StWRI1*, *AsWRI1*, *PtWRI1*, or *CeWRI1*, interestingly the ratio of C18:3 were increased, but the proportions of C18:0 were remarkably decreased in all transgenic leaves compared to transformed control [42]. An et al. [37] reported that the levels (mol%) of C18:1 were increased, but the amounts of C18:0 were decreased in the TAG fractions isolated from leaves expressing *CsWRI1A*, *B*, or *C*. Interestingly, we observed an increase and a decrease in the levels (mol%) of polyunsaturated fatty acid (C18:2 and C18:3) and saturated fatty acids (C16:0, C18:0, and C24:0), respectively, in the TAG fractions induced by the overexpression of *PfWRI1A* or *PfWRI1B* compared to the transformed control (Figure 4C). A similar observation was also reported in *N. benthamiana* leaves expressing *RcWRIA* or *RcWRIB* [52]. Therefore, the overexpression of plant *WRI1s* can alter the ratio of saturated and unsaturated fatty acids in the TAGs accumulated in leaves and they are possibly useful for the production of storage oils with high unsaturated fatty acids in vegetative organs. 

Several *WRI1* orthologs including *PfWRI1* showed their predominant expression in developing seeds or oil-rich non-seed tissues (Figure 2) [58], indicating that they play a crucial role in the deposition of seed storage materials. WRI1s were known to be a key transcriptional regulator, which activates the expression of several genes involved in late steps of glycolysis, fatty acid biosynthesis, and TAG assembly such as *BCCP2*, *Pl-PKβ1*, *PDHE1α*, *enoyl-ACP reductase* (*EAR*), *ACP1*, *KAS1*, and *DGAT1* [29,30,31,38]. The expression of *CsWRIs* caused the upregulation of *BCCP2* and *Pl-PKβ1* in developing seeds of Arabidopsis *wri1-3* [37]. Transient expression of *RcWRI1-A* or *RcWRI1-B* in *N. benthamiana* leaves significantly activated the expression of *ACP1*, *PDHE1α*, *KAS1*, *BCCP2*, *Pl-PKβ1*, and *PlPKα* [52]. We also observed that the expression of *Pl-PKβ1*, *BCCP2*, and *KAS1* was significantly enhanced in *N. benthamiana* leaves by the expression of *PfWRI1s* (Figure 5).

Arabidopsis *WRI1* and its orthologs were reported to be localized in the nucleus [36,37,38,40,59,60], and the fluorescent signals from PfWRI1A:eYFP and PfWRI1B:eYFP were also observed in the nucleus (Figure 3). When the nuclear localization signal (NLS) sequences were searched on the website (https://www.novoprolabs.com/tools/nls-signal-prediction)), the “PRPKRAKRA” motif was predicted in AtWRI1 protein. In the case of PfWRI1A and PfWRI1B, the “VKPKPKRVRAK” motif might be important to be localized in the nucleus. In addition, mutations in any of the three “VYL” residues, which are present in the first AP2 domain of AtWRI1 partially rescued the low seed oil phenotype of *wri1-1* to the wild type and mutations in all three residues failed to restore the fatty acid content of *wri1-1*, indicating that the motif is essential for the function of AtWRI1 [36]. However, Ji et al. [52] reported that both RcWRI1A with the VYL motif and RcWRI1B without the VYL motif were functionally active and restored the wrinkled seed phenotype of *wri1-1*. Although PfWRI1s also contain the “IYL” motif instead of “VYL” (Figure 1A), the role of the “IYL” motif remains to be further investigated in the transcriptional regulation.

## 5. Conclusions

Transcriptome and genetic resources from Perilla [46,47] enabled us to isolate two WRI1 isoforms, *PfWRI1A* and *PfWRI1B* genes from developing Perilla seeds. Ectopic expression upregulated the expression of their target genes in *N. benthamiana* leaves and stimulated storage oil accumulation. These findings can be applied to the production of sustainable and renewable storage oils in leafy biomass to meet the increasing demand for their usage.

## Data Availability

All data supporting the finding of this study are available within the paper and its Supplementary Materials published online.

## Supplementary Materials

The following are available online at https://www.mdpi.com/article/10.3390/plants12051081/s1, Table S1: Primers used in this study. Table S2: The deduced amino acid sequence similarity of WRIs orthologs from various plant species. Figure S1: Nucleotide and deduced amino acid sequences of P. frutescens WRI1A and WRI1B isoforms. Figure S2: Design of PfWRI1A- or PfWRI1B-specific primers. Figure S3: Thin layer chromatography (TLC) of neutral lipids on a silica gel plate developed with a solvent mixture of hexane/ethyl ether/acetic acid (70:30:1, *v*/*v*/*v*).
