# Supplementary material for: Ectopic Expression of Perilla frutescens WRI1 Enhanced Storage Oil Accumulation in Nicotiana benthamiana Leaves
Source: Plants (Basel). 2023 Feb 28;12(5):1081. doi: 10.3390/plants12051081 (PMC10005204; doi:10.3390/plants12051081)
Supplement: Supplementary file 1 [file plants-12-01081-s001.zip › plants-2138600-supplementary Figures.pdf]

A

```
ATGAAGAGAAAGTCTCCCTCACCAGCTTGCTCCTCCTCCTCCTCCTCCTGCTGCATCGAA     60
 M  K  R  K  S  P  S  P  A  C  S  S  S  S  S  S  C  C  I  E      20
TCGCCAACCACGCAGAAAAATGCGCCGCAATCAAAGCCCGACGCCGTCAAACCCAAGCCC    120
 S  P  T  T  Q  K  N  A  P  Q  S  K  P  D  A  V  K  P  K  P      40
AAACGCGTTCGAGCGAAGAAAAACCAGAGCCACAGCAATTCCGCCACCTCCCCTAAATCG    180
 K  R  V  R  A  K  K  N  Q  S  H  S  N  S  A  T  S  P  K  S      60
CGAAGCTCCATCTACAGAGGCGTCACTAGGCATAGATGGACCGGGAGGTATGAAGCTCAC    240
 R  S  S  I  Y  R  G  V  T  R  H  R  W  T  G  R  Y  E  A  H      80
CTCTGGGATAAGACGACATGGAACAGCATTCAGAACAAGCGAGGAAGACAAATCTATTTA    300
 L  W  D  K  T  T  W  N  S  I  Q  N  K  R  G  R  Q  I  Y  L     100
GGAGCTTATGATAATGAGGAAGATGCGGCTCGGACCTACGATCTAGCTGCCCTTAAATAT    360
 G  A  Y  D  N  E  E  D  A  A  R  T  Y  D  L  A  A  L  K  Y     120
TGGGGCCCTGCTACTATCCTCAACTTTCCTGTAGAGGCCTACACCAAAGATCTTGAGGAG    420
 W  G  P  A  T  I  L  N  F  P  V  E  A  Y  T  K  D  L  E  E     140
ATGCAAAAATTGACTAAGGAGGAGTATTTAGCCTCGCTTAGGCGGCGGAGTAGCGGCTTT    480
 M  Q  K  L  T  K  E  E  Y  L  A  S  L  R  R  R  S  S  G  F     160
TCGCGGGGAGTTTCTAAGTATCGCGGTGTAGCAAGGCACCATCACAATGGCCGGTGGGAG    540
 S  R  G  V  S  K  Y  R  G  V  A  R  H  H  H  N  G  R  W  E     180
GCTCGAATTGGGCGTGTTTGCGGAAACAAGTATCTCTACCTAGGAACCTACAGCACCCAA    600
 A  R  I  G  R  V  C  G  N  K  Y  L  Y  L  G  T  Y  S  T  Q     200
GAGGAAGCAGCAGCAGCATATGATATGGCAGCAATAGAATTCAGAGGTCCAAACGCTGTA    660
 E  E  A  A  A  A  Y  D  M  A  A  I  E  F  R  G  P  N  A  V     220
ACAAATTTCGACGTAAGTAATTATGCAGATAAGTTAAAGAAATTCCTACCGGAGGTGCAT    720
 T  N  F  D  V  S  N  Y  A  D  K  L  K  K  F  L  P  E  V  H     240
GTCAGACAGGAAGAGGTTCATGTGAAGCAGGAAACCAATTCTGTTATCCCTGATGAAGAA    780
 V  R  Q  E  E  V  H  V  K  Q  E  T  N  S  V  I  P  D  E  E     260
GTACAAGCAGTTGAAGCACAAGATAATCAAGAATATCCAAACCAGTTTGCTCAAACAAGT    840
 V  Q  A  V  E  A  Q  D  N  Q  E  Y  P  N  Q  F  A  Q  T  S     280
ACACCTGAGCCAGAATCGAAAGATTCCATTGAGTCGGAAGACATACTCGTCATGGACCTG    900
 T  P  E  P  E  S  K  D  S  I  E  S  E  D  I  L  V  M  D  L     300
TCCGAGGAGCACGAGAACCCTTGGGATCTCTGTCTAGACACTGTATTCAATATCCTCCCA    960
 S  E  E  H  E  N  P  W  D  L  C  L  D  T  V  F  N  I  L  P     320
ATCCCTGACCTTCCTCTGGGTAAAGCCTCCGAGGTATTCGATTACCATGGTTTTGAGGAC   1020
 I  P  D  L  P  L  G  K  A  S  E  V  F  D  Y  H  G  F  E  D     340
GATATTGAATGCATCTTCGACGAGCCATTAGATGATAATGAGAACTTCCAGAATGCAATG   1080
 D  I  E  C  I  F  D  E  P  L  D  D  N  E  N  F  Q  N  A  M     360
CTTGGATGCCAGGTTGATGCAGATGCTTTGGTATCTCAAAACTTGAAAGGGACGAATTCT   1140
 L  G  C  Q  V  D  A  D  A  L  V  S  Q  N  L  K  G  T  N  S     380
CCTTCTTCTCCTTCTTTCGTCGCCATTATCATCGACCATATCAGCTTGCAGCAACATGTAA   1200
 P  S  S  P  S  S  S  P  L  S  S  T  I  S  A  C  S  N  M  *     400
```

B

```
ATGAAGAGAAAGTCTCCCTCACCAGCTTGCTCCTCCTCCTCCTCCTCCTCCTGCTGC   60
 M  K  R  K  S  P  S  P  A  C  S  S  S  S  S  S  S  S  C  C   20
ATCGAATCGCCAACCGCGCAGAAAAATGCGCCGCAATCAAAGGCCGACGCCGTCAAACCC  120
 I  E  S  P  T  A  Q  K  N  A  P  Q  S  K  A  D  A  V  K  P   40
AAGCCCAAACGCGTTCGAGCGAAGAAAAACCAGAGCCACAGCAATTCCGCCACCTCCCCT  180
 K  P  K  R  V  R  A  K  K  N  Q  S  H  S  N  S  A  T  S  P   60
AAATCGCGCAGCTCCATCTACAGAGGCGTCACTAGGCATAGATGGACTGGGAGGTACGAA  240
 K  S  R  S  S  I  Y  R  G  V  T  R  H  R  W  T  G  R  Y  E   80
GCTCACCTCTGGGATAAGACGACATGGAACAGCATTCAGAACAAGCGAGGAAGACAAATC  300
 A  H  L  W  D  K  T  T  W  N  S  I  Q  N  K  R  G  R  Q  I  100
TATTTAGGAGCTTATGATAATGAGGAAGATGCGGCTCGGACCTACGATCTAGCTGCCCTT  360
 Y  L  G  A  Y  D  N  E  E  D  A  A  R  T  Y  D  L  A  A  L  120
AAATATTGGGGCCCTGCTACTATCCTCAACTTTCCTGTAGAGGCCTACACCAAAGATCTT  420
 K  Y  W  G  P  A  T  I  L  N  F  P  V  E  A  Y  T  K  D  L  140
GAGGAGATGCAAAAATTGACTAAGGAGGAGTATTTAGCCTCGCTTAGGCGGCGGAGTAGC  480
 E  E  M  Q  K  L  T  K  E  E  Y  L  A  S  L  R  R  R  S  S  160
GGCTTTTCGCGGGGAGTTTCTAAGTATCGCGGTGTAGCAAGGCACCATCACAATGGCCGG  540
 G  F  S  R  G  V  S  K  Y  R  G  V  A  R  H  H  H  N  G  R  180
TGGGAGGCTCGAATTGGGCGCGTTTGCGGAAACAAGTATCTCTACCTAGGAACCTACAGC  600
 W  E  A  R  I  G  R  V  C  G  N  K  Y  L  Y  L  G  T  Y  S  200
ACCCAAGAGGAAGCAGCAGCAGCATATGATATGGCAGCAATAGAATTCAGAGGTCCAAAC  660
 T  Q  E  E  A  A  A  A  Y  D  M  A  A  I  E  F  R  G  P  N  220
GCTGTAACAAATTTCGATGTAAGTAATTATGCAGATAAGTTAAAGAAATTCCTACCGGAG  720
 A  V  T  N  F  D  V  S  N  Y  A  D  K  L  K  K  F  L  P  E  240
GTGCAAGTCAGACAGGAAGAGGTCCATGTGAAGCAGGAAACCAATTCTGTTACCCTTGAT  780
 V  Q  V  R  Q  E  E  V  H  V  K  Q  E  T  N  S  V  T  L  D  260
GAAGAAGTACAAGCAGTTAAAGCACAAGATAATCAAGAATATCCAAACCAGTTTGCTCAA  840
 E  E  V  Q  A  V  K  A  Q  D  N  Q  E  Y  P  N  Q  F  A  Q  280
ACAAGTACACCTGAGCCAGAATCGAAAGATTCCATTGAGTCGGAAGACATACTCGTCATG  900
 T  S  T  P  E  P  E  S  K  D  S  I  E  S  E  D  I  L  V  M  300
GACCTGTCCGAGGAGCACGAGCACCCTTGGGATCTCTGTCTAGACACTGTATTCAATATC  960
 D  L  S  E  E  H  E  H  P  W  D  L  C  L  D  T  V  F  N  I  320
CTCCCAATCCCTGACCTTCCTCTGGGTAAAGCCTCCGAGGTATTCGATTACCATGGTTTT 1020
 L  P  I  P  D  L  P  L  G  K  A  S  E  V  F  D  Y  H  G  F  340
GAGGACGATATTGAATGCATCTTCGACGAGCCATTAGATGATAATGAGAACTTCCAGAAT 1080
 E  D  D  I  E  C  I  F  D  E  P  L  D  D  N  E  N  F  Q  N  360
GCAATGCTTGGATGCCAGGTTGATGCAGATGCTTTGGTATCTCAAAACTTGAAAGGGACG 1140
 A  M  L  G  C  Q  V  D  A  D  A  L  V  S  Q  N  L  K  G  T  380
AATTCTCCTTCGTCGTCGCCATTATCATCGACCATATCAGCTTGCAGCAACATGTAA    1197
 N  S  P  S  S  S  P  L  S  S  T  I  S  A  C  S  N  M  *     399
```

Supplementary Figure S1. Nucleotide and deduced amino acid sequences of *P. frutescens WRI1A* and *WRI1B* isoforms

The conserved AP2/ERF DNA binding motifs and transcriptional activation motif [36] in WRI1 are shown in green and yellow backgrounds, respectively. The non-conserved nucleotide and amino acid sequences between *PfWRI1A* and *PfWRI1B* are shown in dark-gray and light-gray backgrounds, respectively.

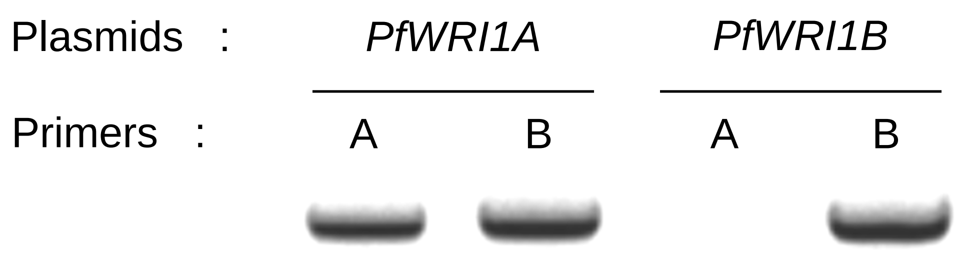

Supplementary Figure S2. Design of *PfWRI1A*- or *PfWRI1B*- specific primers.

Plasmids harboring PfWRI1A or *PfWRI1B* were used in PCR analysis as templates. The designed A- and B-specific primers are shown in Supplementary Figure S1.

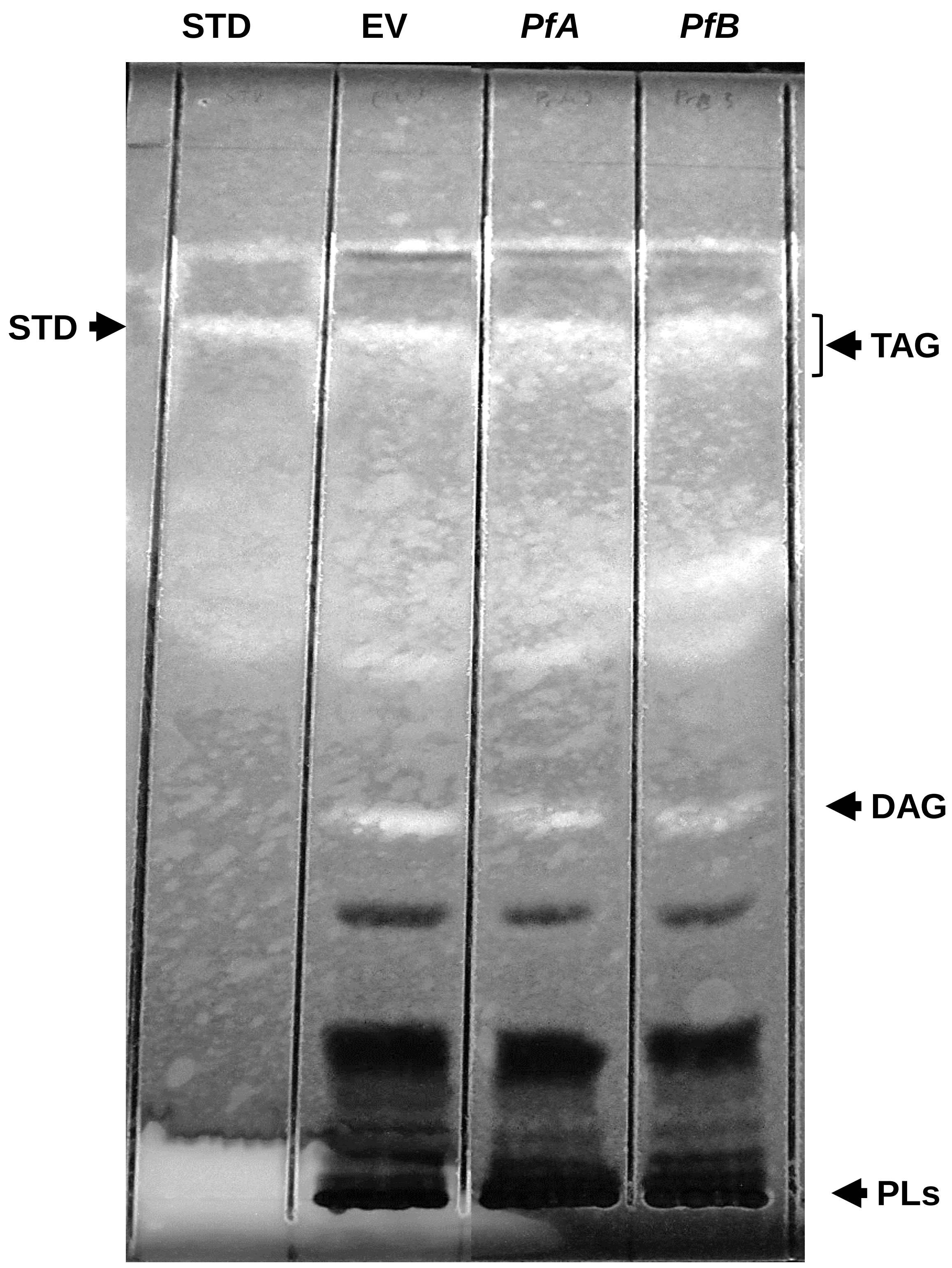

Supplementary Figure S3. Thin layer chromatography (TLC) of neutral lipids on a silica gel plate developed with a solvent mixture of hexane/ethyl ether/acetic acid (70:30:1, v/v/v).

TAG, triacylglycerol; DAG, diacylglycerol; PLs, polar lipids; EV, empty vector; *PfA*, *PfWRI1A; PfB*, *PfWRI1B*; STD, glycerol triheptadecanoate (C17:0).
